# The Synthesis and Evaluation of Porous Carbon Material from Corozo Fruit (*Bactris guineensis*) for Efficient Propranolol Hydrochloride Adsorption

**DOI:** 10.3390/molecules28135232

**Published:** 2023-07-05

**Authors:** Dison Stracke Pfingsten Franco, Jordana Georgin, Claudete Gindri Ramos, Salma Martinez Eljaiek, Daniel Romero Badillo, Anelise Hoch Paschoalin de Oliveira, Daniel Allasia, Lucas Meili

**Affiliations:** 1 Department of Civil and Environmental, Universidad de la Costa, CUC, Calle 58# 55-66, Atlántico, Barranquilla 080002, Colombiaseljaiek3@cuc.edu.co (S.M.E.); dbadillo@cuc.edu.co (D.R.B.); 2 Graduate Program in Environmental Engineering, Federal University of Santa Maria, Santa Maria 97105-900, RS, Brazil; dallasia@gmail.com; 3 Graduate Program in Chemical Engineering, Federal University of Santa Maria, Santa Maria 97105-900, RS, Brazil; anelisehpo@gmail.com; 4 Process Laboratory, Technology Center, Federal University of Alagoas, Maceió 57072-870, AL, Brazil; lucas.meili@ctec.ufal.br

**Keywords:** batch adsorption, residue, activated carbon, emergent pollutants, propranolol, model

## Abstract

This study explores the potential of the corozo fruit (*Bactris guineensis*) palm tree in the Colombian Caribbean as a source for porous carbon material. Its specific surface area, pore volume, and average pore size were obtained using N_2_ adsorption/desorption isotherms. The images of the precursor and adsorbent surface were obtained using scanning electron microscopy (SEM). Fourier transform infrared (FTIR) spectra were obtained to detect the main functional groups present and an X-ray diffraction analysis (XRD) was performed in order to analyze the structural organization of the materials. By carbonizing the fruit stone with zinc chloride, a porous carbon material was achieved with a substantial specific surface area (1125 m^2^ g⁻^1^) and pore volume (3.241 × 10−^1^ cm^3^ g⁻^1^). The material was tested for its adsorption capabilities of the drug propranolol. The optimal adsorption occurred under basic conditions and at a dosage of 0.7 g L⁻^1^. The Langmuir homogeneous surface model effectively described the equilibrium data and, as the temperature increased, the adsorption capacity improved, reaching a maximum of 134.7 mg g⁻^1^ at 328.15 K. The model constant was favorable to the temperature increase, increasing from 1.556 × 10^−1^ to 2.299 × 10^−1^ L mg^−1^. Thermodynamically, the adsorption of propranolol was found to be spontaneous and benefited from higher temperatures, indicating an endothermic nature (12.39 kJ mol⁻^1^). The negative ΔG^0^ values decreased from −26.28 to −29.99 kJ mol^−1^, with the more negative value occurring at 328 K. The adsorbent material exhibited rapid kinetics, with equilibrium times ranging from 30 to 120 min, depending on the initial concentration. The kinetics data were well-represented by the general order and linear driving force models. The rate constant of the general order model diminished from 1.124 × 10^−3^ to 9.458 × 10^−14^ with an increasing concentration. In summary, the leftover stone from the *Bactris guineensis* plant can be utilized to develop activated carbon, particularly when activated using zinc chloride. This material shows promise for efficiently adsorbing propranolol and potentially other emerging pollutants.

## 1. Introduction

The existence of rising micropollutants in the environment, which are discharged into the environment in a variety of societal sectors, including residential, commercial, and, most significantly, hospital effluents, is one of the most serious environmental challenges today [1,2]. Furthermore, the United Nations (UN) is aiming for green development, lobbying for the adoption of environmental legislative laws on global water resource contamination, and enabling research on these ecological toxins [1]. There is a serious issue with the category of pharmaceuticals included in the emerging contaminants, since environmental regulations are lacking despite the ongoing discharges of effluents containing these contaminants [3,4,5]. It should be emphasized that these medications can be extremely harmful to aquatic life even in low quantities, especially over a long period [6,7,8,9].

Propranolol hydrochloride (C_16_H_21_NO_2_.HCl), a medicine used to treat hypertension, is widely used around the world, where a significant percentage of it is eliminated in the urine and not absorbed by the body [10]. When taken, propranolol has a long duration and is highly persistent in the environment. Its main metabolite is 4-hydroxy propranolol [11,12]. Conventional effluent treatment techniques remove only a portion of this contaminant [10], with the remainder being dumped into bodies of water, where it is very toxic to a variety of organisms, including *S. vacuolatus* algae [13]. For example, a study conducted by Santos et al. [14] on the effluent wastewaters from four different hospitals located in Coimbra (Portugal) showed the presence of different drugs, among them propranolol, in all the hospital samples analyzed and also in the WWTP influent and effluent, where the removal efficiency for β-blockers was <17%. In another study carried out by Grover et al. [15], a full-scale, granular, activated carbon plant treating a WWTP effluent was evaluated in terms of its removal efficiency of pharmaceuticals. A higher removal efficiency (84–99%) was observed for mebeverine, indomethacine, and diclofenac, while carbamazepine and propranolol displayed a much lower removal efficiency (17–23%). 

Brazilian surface water, drinking water, and effluents from sewage and water treatment facilities have all been discovered to contain propranolol [14,15]. The most recent studies by Starling et al. [16] and Peña-Guzmán et al. [17] found propranolol in river waters in the north of Brazil (26 ng L^−1^), and in the drinking (7000 a 50,000 ng L^−1^), surface (3.1 a 43.9 ng L^−1^), effluents (39.6 a 45.5 ng L^−1^), and affluents (3.86 a 15.3 ng L^−1^) of sewage treatment plant (STP) waters in many countries in Latin America. Numerous methods have been investigated as a result of this issue, mostly for the removal and degradation of this chemical [1,16,17,18,19], for example, photolysis, photo-Fenton, photocatalysis, ozonation, nanofiltration, and adsorption [18,19,20]. Adsorption is a popular method for eliminating contaminants from water and wastewater due to its effectiveness and low cost [21,22,23,24]. Adsorption has the advantage of being able to use new adsorbents produced from plant leftovers [25,26]. Plant leftovers, such as those produced by agricultural activities or plant-based industries, can be used to produce activated carbon at a cheap cost and in a sustainable manner [27]. These carbonaceous materials, which are mostly made of carbon, often have an excellent pore formation and a high surface area, which are essential characteristics of an effective adsorbent [28].

Native to the Caribbean and Central America, the *Bactris guineensis* palm is a fruit tree. Economically, this fruit tree is very valuable [29]. Additionally, known by its popular name, Corozo’s small, oblong fruits can be used to make drinks, jellies, and even wines, in addition to being consumed in their natural state [30,31]. A single plant produces 30 kg of fruit per year, resulting in an output of 750 kg ha^−1^ annually [29], producing significant amounts of leftover biomass, which equates to seeds and bark. Research has indicated that its black pulp has antioxidant capabilities [30,32]. The utilization of its residual biomass as an adsorbent material and its potential application in the removal of emerging contaminants have not been reported in any studies.

In this study, the residual biomass of *Bactris guineensis* fruit, known as stone, was used to generate activated carbon using zinc chloride (ZnCl_2_), which was then employed as an adsorbent to extract propranolol hydrochloride. The original and carbonized materials were thoroughly analyzed, utilizing various techniques to assess their qualities. The BET surface area and porosity values were obtained using nitrogen isotherms. The main functional groups were obtained using Fourier transform infrared (FTIR) spectra, the structural arrangement was analyzed using the X-ray diffraction method, and the surface micrography was obtained using SEM. Following that, activated carbon was used to remove propranolol hydrochloride and studies were undertaken to identify the best adsorbent dosage and pH. Kinetic and isothermal experiments were also performed and mathematical models were used to fit the experimental data.

## 2. Results and Discussion

### 2.1. Characterization Results

The final yield and surface area of the material were significantly impacted by the conversion of lignin and cellulose into volatile material during the pyrolysis stage [33]. The resulting carbonaceous skeleton, composed mainly of carbon, exhibited a yield of approximately 27%, consistent with previous studies using a 1:1 proportion of ZnCl_2_. The four biomasses examined in this investigation were Jabuticaba residues [34], açai residues [35], Hovenia dulcis fruits [27], and cassava peels [36]. The authors reported a yield of about 64 % when KOH was used as the activating agent [37]. The carbonization with ZnCl_2_ resulted in desirable textural properties for an adsorbent, including a high surface area (S_BET_ = 1125.4 m^2^ g^−1^) and well-developed pores (Vp = 3.241 × 10^−1^ cm^3^ g^−1^; Dp = 2.321 nm) (according to Figure S1). One of the important points that defines the adsorption capacity of a material is its surface area. Hence, the utilization of ZnCl_2_ for carbonization resulted in certain factors that could limit the performance of the material against PROP. 

**Figure 1:**
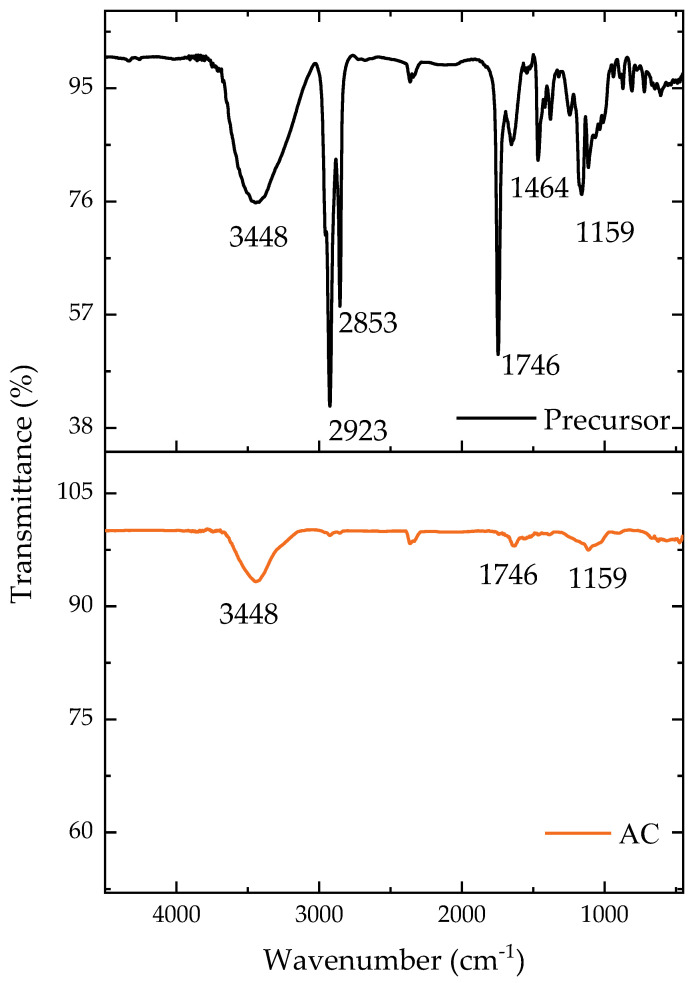
Transmittance according to the wavenumber for the precursor material and AC.

The main functional groups present on the material surface can be identified in Figure 1. The majority of the groups were still present following the carbonization, but with a diminished intensity, as can be seen. The O-H bonding region at 3448 cm^−1^ [38] remained after the carbonization but with a reduced intensity, while the CH binding at 2923 cm^−1^, found only in the precursor material, was consumed during the pyrolysis step [39]. The disappearance of these functional groups indicated the loss of volatile material during pyrolysis [40]. The asymmetric vibrations of CH_2_ in the precursor were found at 2853 cm^−1^ [41]. Both the precursor and pyrolyzed materials had CO groups (1746 cm^−1^), generally attributed to ketones [42]. Aromatic rings were present in the precursor material at 1464 cm^−1^ [40,43]. Finally, the CO groups (1159 cm^−1^) were found in both the precursor and pyrolyzed materials with oxygen (C-O) [42]. These spectra confirm that the carbonization with ZnCl_2_ reduced the heterogeneity of the functional groups to some extent.

**Figure 2:**
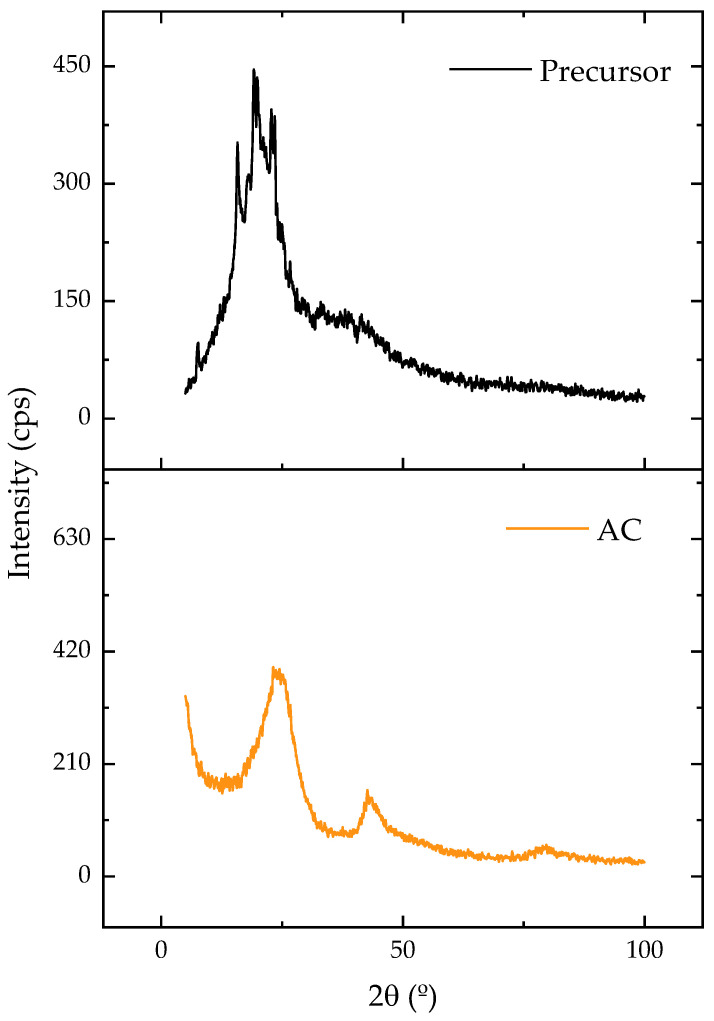
XRD patterns for precursor and AC.

Figure 2 displays the X-ray diffraction patterns of the original and carbonized samples. Amorphous carbon was linked to the presence of a broad diffraction band between 15 and 30 [44]. Following the carbonization, this band showed a reduction in its width and an increase in its intensity, which could be attributed to the formation of a more crystalline and ordered structure. Despite being disorganized and irregular, amorphous arrangements offer empty spaces that can be occupied by adsorbate molecules, which enhances adsorption [28].

**Figure 3:**
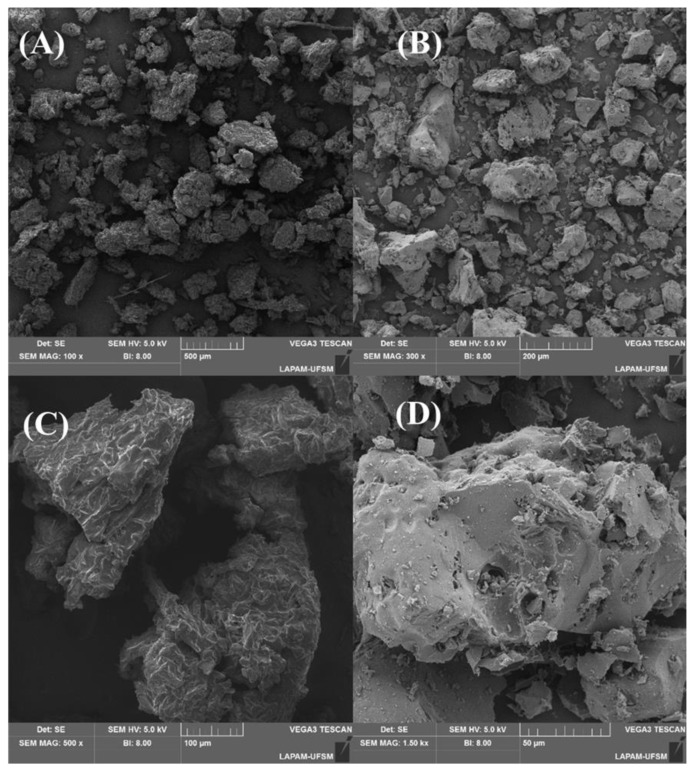
SEM images of precursor material (**A**,**C**), and activated carbon (**B**,**D**).

The application of a high temperature and activation brought about significant morphological alterations on the material’s surface, as depicted in Figure 3. Figure 3A,C show that the surface was composed of various particles that exhibited diverse sizes and shapes, a feature that remained consistent after the carbonization. As for the surface of the original material (C), cracks were evident that extended throughout the entire particle. Some irregularities and random protrusions could also be seen. Following the carbonization (D), the surface became more irregular and smoother, but with the addition of cavities of varying sizes that are crucial for accommodating adsorbate molecules. The morphological structures of materials made from lignin and cellulose are typically irregular and disorganized, resembling the structure of fruit waste [34,44,45], forest seed species [46,47,48], and tree bark [49,50,51]. These irregularities are evenly distributed and contain cavities and voids that can significantly enhance adsorption [52].

### 2.2. Adsorbent Dosage and Solution pH Optimization

**Figure 4:**
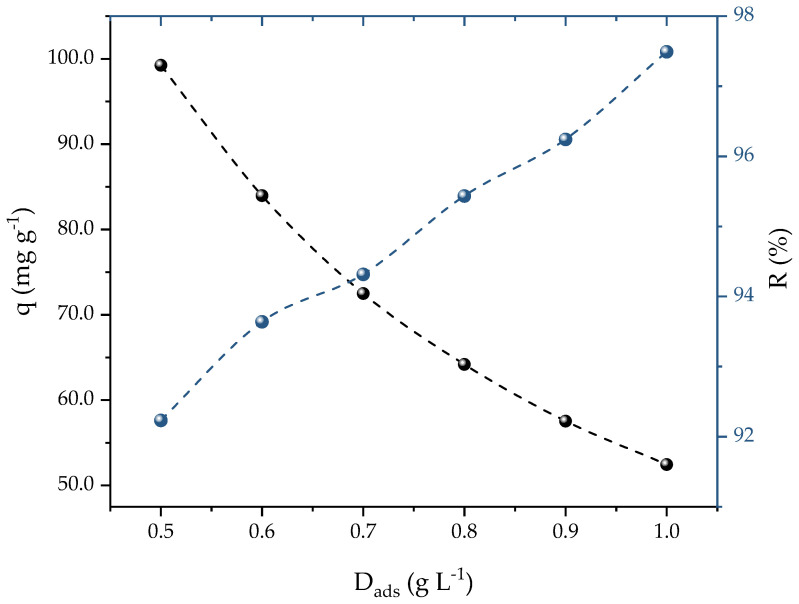
Effect of AC dosage on PROP adsorption (V = 25 mL, T = 298.15 K, natural solution pH, C_0_ = 25 mg L^−1^, and t = 120 min).

A concentration of 25 mg L^−1^ of PROP was used to calculate the ideal dosage of activated carbon (AC) for the adsorption of the PROP. Figure 4 shows that, as the AC dosage increased from 0.5 to 1 g L^−1^, two different effects were generated: (i) a decrement of the adsorption capacity (99 to 54 mg g^−1^); and (ii) an increment of the removal efficiency (92 to 97%). It should be noted that a plateau in the percentages of the removal and adsorption capacities was not found, which can be explained due to the range of dosages studied. This could be a problem if the aim was to determine the dosage which presented the highest removal. However, in this case, the aim was an ideal dosage that presented a balance between the adsorption capacity and the percentage of removal that was found when the theoretical curves crossed each other to obtain the optimal dosage. In this case, this occurred at 0.7 g L^−1^, which yielded satisfactory values for both the capacity (72.48 mg g^−1^) and removal (94%). Therefore, for future studies, 0.7 g L^−1^ of AC was used. In a different investigation, iron nanocomposite in ionic liquid was used as an adsorbent for PROP removal; Ali et al. [53] observed that increasing the dosage also increased the removal efficiency, reaching 90% at 0.5 g L^−1^.

**Figure 5:**
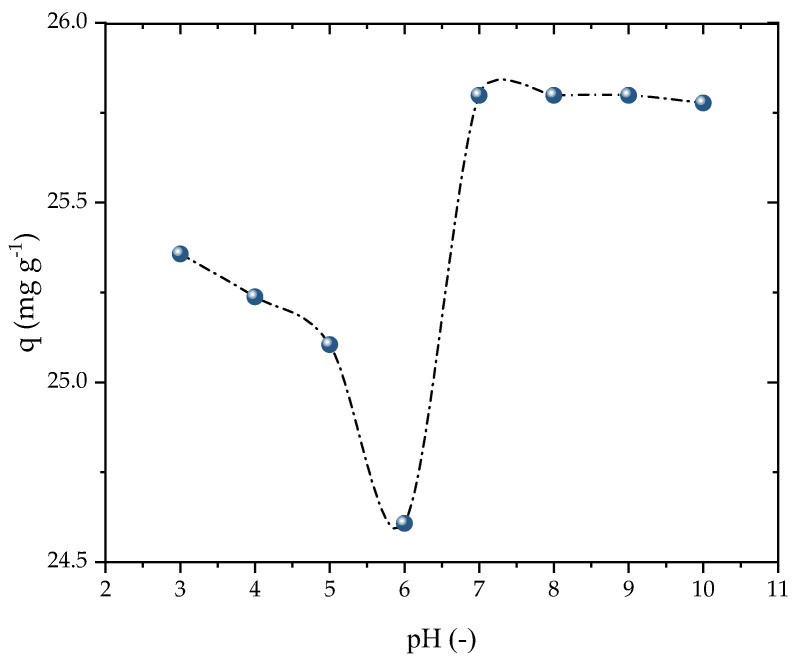
Effect of pH on PROP adsorption (V = 25 mL, T = 298.15 K, D_ads_ = 0.7 g L^−1^, C_0_ = 25 mg L^−1^, and t = 120 min).

The impact of the initial solution pH was evaluated in the range of 3 to 10, as presented in Figure 5. The adsorption capacity marginally decreased when increasing the pH from 3 to 6, while it was enhanced as the pH was raised from 6 to 10. However, the adsorbent exhibited a stable performance in terms of its adsorption capacity at all pH levels, making it highly suitable for potential full-scale applications. For the subsequent kinetic and isothermal studies, basic conditions similar to the natural state of the solution were employed. This outcome was consistent with the findings of Ali et al. [53], which demonstrated that the adsorption capacity of PROP increased with the pH up to nearly 9, before decreasing again. The charges of the β-blockers in the solution were primarily positive because of PROP’s consistent acidity as a secondary amine (pK_a_ = 9.5), which favors adsorption at a high pH. Because of the amine’s limited solubility and the hydrolysis at pH levels above 8, adsorption above this range is impracticable [54]. Thus, the pH solution was chosen to be 8 for the investigations that followed.

### 2.3. Isothermal Experiments and Estimative of the Adsorption’s Thermodynamic Proprieties

**Figure 6:**
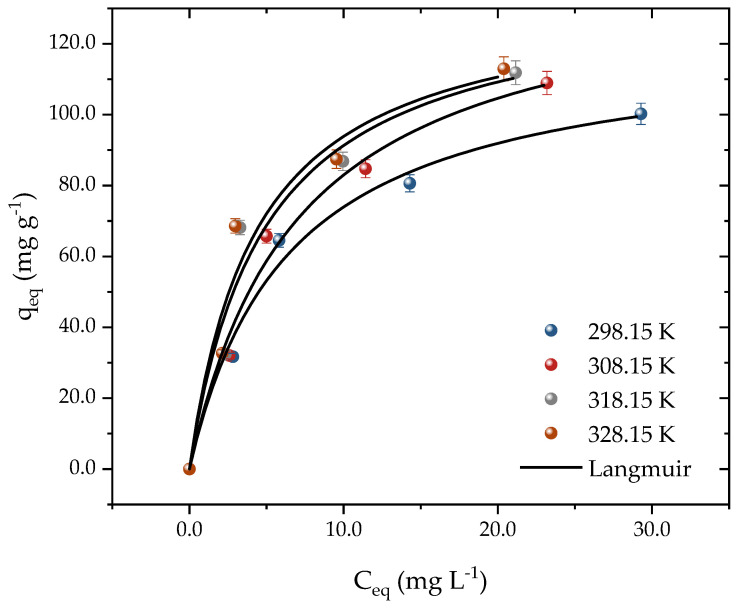
Equilibrium adsorption data and Langmuir model prediction for PROP adsorption on activated carbon according to the system temperature (T = 298.15, 308.15, 318.15, and 328.15 K, pH = 10, D_ads_ = 0.7 g L^−1^, and t = 300 min).

Equilibrium curves were constructed at temperatures from 298 to 328 K through the isothermal studies, as depicted in Figure 6. The experimental data acquired in this research shed light on the interaction mechanisms between activated carbon (AC) and propranolol (PROP) molecules. The Langmuir model was employed to analyze the adsorption isotherm data and estimate the thermodynamic parameters. The outcomes of the thermodynamic analysis furnished essential information on the energetics of the adsorption process and the nature of the interaction between the adsorbent and the adsorbate. The concentration of PROP was varied from 25 to 100 mg L^−1^ to construct the curves, all of which exhibited favorable L-shaped adsorption behavior [55]. At 298 K, the adsorption capacity rose from 31 to 100 mg g^−1^ as the PROP concentration increased. The effect of the temperature on the drug adsorption was found to be significant, with the capacity changing from 100 to 112 mg g^−1^ when the temperature was increased from 298 K to 328 K. This indicated that higher temperatures were more conducive to drug adsorption onto the adsorbent surface. The adsorbent exhibited a good performance even at room temperature, which is advantageous for real-world applications, as it requires less energy consumption. This is explained by the driving force gradient, which rose as the adsorbate concentration did [56]. Even though there is little research comparing the adsorption of PROP to other developing contaminants, several studies have shown outcomes that are comparable to those of this one. For instance, when PROP was adsorbed in bentonite clay at a temperature increase from 293 to 313 K, the capacities increased from 0.298 to 0.426 mmol g^−1^ [1].

Table 1 shows the findings obtained for the fitted isothermal models. The Langmuir isotherm had the greatest values that were closest to 1 when the determination coefficients (R^2^) and adjusted determination coefficients (R_2adj_) were examined. Corroborating these results, the lowest values of ARE (%) and MSR (mg g^−1^)_2_ were achieved using the Langmuir. Added to this, the predicted adsorption capacity at equilibrium was close to the experimental capacity. The adsorption capacity at equilibrium increased from 121 to 134 mg g^−1^ with an increasing temperature in the system. In addition, the Langmuir coefficient increased from 0.15 to 0.22 with an increasing temperature, indicating that the activated carbon and PROP molecules had a higher affinity at higher temperatures. A good fit to the Langmuir isotherm suggested that the material’s surface was uniform and that adsorption occurred in monolayers [57,58]. Various studies have used carbonaceous materials and the Langmuir model to accurately depict adsorption, as seen in the literature [1,53,57,59,60].

It may be concluded that the residual stone of carbonized *Bactris guineensis* fruits has a high potential for use in effluents containing PROP after examining the maximal capacity achieved using the Langmuir model (134.7 mg g^−1^) and comparing it with other studies in the literature. For instance, a capacity of 0.468 mmol g^−1^ for the concentration range of 0.05 to 3 mmol L^−1^ of PROP was observed when employing bentonite clay as an adsorbent [1]. When utilizing Montmorillonite as an adsorbent and changing its dosage from 0.5 to 80 mg L^−1^, the highest capacity of 6.2 × 10^5^ mol g^−1^ was attained [54]. Ali and collaborators [53] reported a capacity of 105.26 mg g^−1^ when employing liquid iron nanocomposite. The authors obtained a capacity of 6.67 μmol g^−1^ for a concentration range of 0.8 to 30 mg L^−1^ using corn husk biochar [61]. Using p-doped mesoporous carbon, Paixão and associates [62] reported a capacity of 287 mg g^−1^. In contrast, Feizi et al. [63], in a fixed bed system with magnetic tire char, reached a capacity of 22.58 mg g^−1^. 

The thermodynamic parameters obtained from the isothermal studies are presented in Table 2. The Langmuir model constant was used to estimate the equilibrium constant and thermodynamic parameters, as it was found to be the most suitable model. As the temperature rose from 298 to 328 K, the Ke values increased from 4.035 to 5.962 × 10^4^, indicating a stronger adsorption affinity at higher temperatures. Furthermore, the negative ΔG^0^ values decreased from −26.28 to −29.99 kJ mol^−1^, with the more negative value occurring at 328 K, suggesting that the adsorption of PROP was spontaneous at this temperature. The endothermic nature of the thermodynamic process (∆H^0^ = +12.39 kJ mol^−1^) observed in this study was consistent with the isothermal studies. The magnitude of ∆H^0^ indicates the presence of strong physical forces, such as electrostatic attraction, implying that the main process in this study was physisorption, which is reversible and allows for desorption. Moreover, the positive value of ΔS^0^ (0.1290 kJ mol^−1^ K^−1^) suggests a high affinity between PROP and AC. Similar endothermic behavior has been observed in previous studies involving propranolol hydrochloride adsorption on thermally treated bentonite clay [1].

### 2.4. Propranolol Adsorption Kinetics

**Figure 7:**
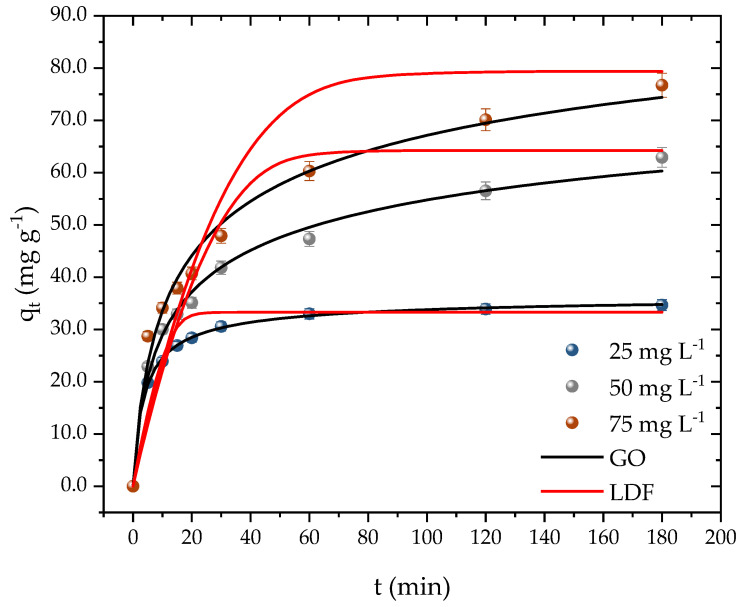
PROP adsorption kinetic curves on activated carbon (T = 298.15 K, D_ads_ = 0.7 g L^−1^, pH = 10, and V = 25 mL).

Kinetic time is a crucial parameter, as it determines the time required for the adsorbent surface to be fully occupied by the adsorbate molecules. Adsorbents with fast kinetics are desirable as they indicate a high affinity with the pollutant and can result in cost savings during the operational process. Figure 7 illustrates that the equilibrium time was dependent on the initial concentration of PROP. At the lowest concentration (25 mg L^−1^), equilibrium was achieved in 30 min with an adsorption capacity of 30 mg g^−1^. For the second concentration (50 mg L^−1^), the system reached equilibrium after 120 min, attaining a capacity of 56 mg g^−1^. The system took 180 min to reach equilibrium at the highest concentration, resulting in an adsorption capacity of 76 mg g^−1^.

Despite the difference in the kinetic times, it can be inferred that, regardless of the concentration, the adsorbent developed had a high affinity with PROP and a relatively fast kinetic time. Instances of both fast and slow kinetics were found while examining the kinetic behavior of PROP by other adsorbents in the literature. A high affinity of the adsorbent with the adsorbate was confirmed when authors examined the adsorption of PROP on montmorillonite and found that, during the first minute, roughly 96 percent of the medication had already been removed [54]. In river water samples that had been artificially contaminated with PROP, authors, who were already utilizing Na-mica-4 and C18-mica-4, took 24 h to achieve a 97 percent clearance [64]. Finally, authors have reported removals of 68 and 88 percent in durations of 90 min [65] and 180 min [66], respectively, while utilizing granular activated carbon. The results attained from the kinetics tests can also be compared with other methods employed in removal/treatment methods. In this case, the adsorption capacity corresponded to percentages of removal of 93.36, 89.58, and 70.35 % for the initial concentrations of 25, 50, and 75 mg L^−1^, respectively. Gao and collaborators [19] reported a percentage of removal of 94.9 % of PROP in 10 min with an initial concentration of 5.19 mg L^−1^ when employing the UV/persulfate method. Remache et al. [67] investigated the degradation of propranolol using the Fenton method, where they reported a percentage of 95 % in 10 min for an initial concentration of 61.7 mg L^−1^. The results from the different techniques showed that the propranolol can be removed or degraded. The UV and Fenton methods presented quicker kinetics in comparison to the adsorption, which took around 180 min. However, these results came at cost of generating further molecules, which demanded another treatment. As for the adsorption method, the molecule tended to retain stable at the surface of the adsorbent and can be further recovered whiteout undergoing a chemical change. 

Table 3 displays the fitting outcomes of the pseudo-first-order, pseudo-second-order, general order, and linear driving force model (LDF) to the kinetic data. The general order model demonstrated the highest determination coefficients (R^2^; R^2^_adj_) among the four models, followed by the pseudo-second-order model, while the pseudo-first-order model showed the lowest values. Moreover, the general order model also exhibited the lowest values of ARE (%) and MSR (mg g^−1^)^2^, while the pseudo-first-order model showed the highest values. These findings indicate that the adsorption rate primarily relied on the adsorbate concentration and the active sites’ effective number on the adsorbent surface. Consequently, it is possible to establish a general law equation for the adsorption rate [68,69]. The LDF is considered to be one of the simplest models utilized to obtain information related to adsorbent diffusivity [70,71]. The other models do not provide this information. The model showed lower coefficients compared to the other models. Nevertheless, it was observed that the diffusivity decreased from 5.2 to 3.9 with an increase in the PROP concentration. Similar outcomes were obtained with the model coefficient, which decreased from 5.1 to 3.9. These results corroborate other studies that have also used the model for the adsorption of emerging pollutants [35,52,72].

### 2.5. Adsorption Regeneration Results

**Figure 8:**
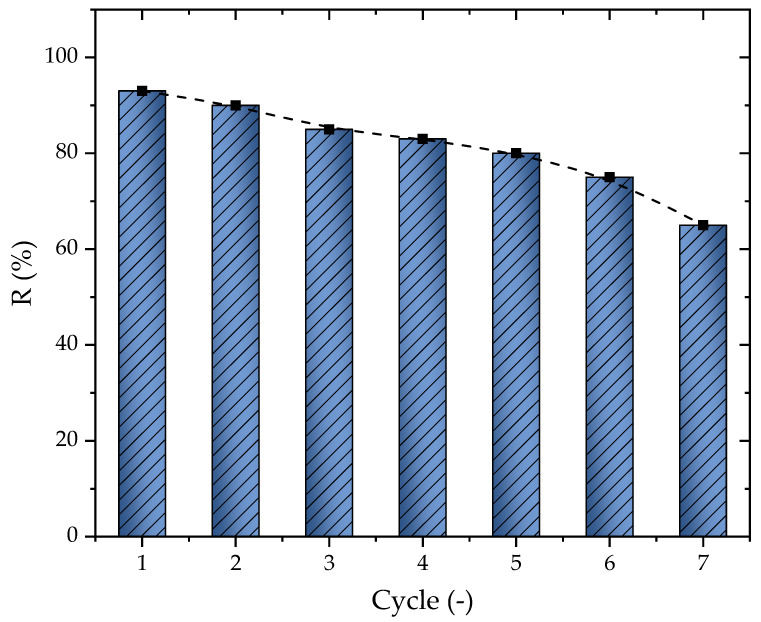
Percentage of removal change according to the cycle number (T = 298.15 K, D_ads_ = 0.7 g L^−1^, pH = 10, and V = 25 mL).

In addition to the results obtained for the adsorption capacity, the percentage of removal change according to the number of cycles was another important aspect of the adsorption study. The regeneration was conducted through solvent extraction and is presented here in Figure 8. The first cycle presented a removal of 93%, which tended to slowly decrease after each cycle until reaching 65% at cycle seven, presenting an average of a 4.67% loss per cycle. The diminishing of the percentage of removal was directly related to the solvent and other effects such as the deactivation of the AC sites or even the steric impendence that occurred depending on the pore diameter [72,73]. Overall, it is possible to conclude that the material could be used until six cycles, where the percentage of removal stayed at 74.9%

### 2.6. Possible Adsorption Mechanism

**Figure 9:**
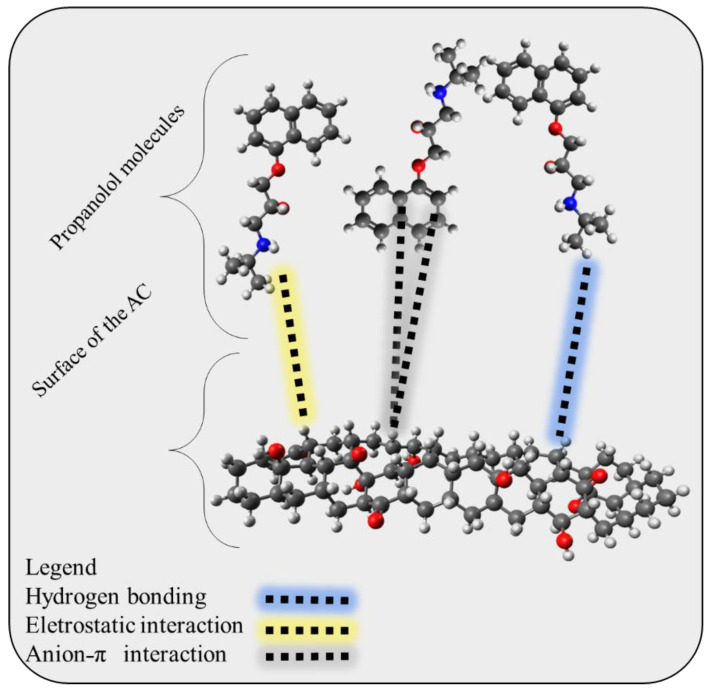
Proposed adsorption mechanism that may occur at the surface of the activated and the adsorbed propranolol molecules.

Once the adsorbent was characterized and its standard enthalpy change was determined, an adsorption mechanism could be proposed. The minimum adsorption surface should first be identified by examining the FT-IR results. The C-H, C=O, OH (phenolic), and aromatic rings were discovered to be the traditional groups for activated biochar. Moreover, for solutions with a pH greater than 6.56, the adsorbent surface was negatively charged (refer to Figure S2). Another crucial element was the adsorbate’s speciation, with PROP having two states: neutral and protonated, because of the amine group (refer to Figure S3). Last but not least, the bond was a physical interaction, as shown by the thermodynamic magnitude ∆H^0^. It is conceivable to propose an adsorption mechanism by taking into account all of these system components, as shown in Figure 9. PROP is anticipated to bind to surfaces via hydrogen bonds, Van der Waals interactions, or ion–ion interactions [74,75]. Overall, this comprehensive approach to characterizing the adsorbent and adsorbate can provide important insights into the adsorption mechanism, which may have implications for the development of more effective adsorbents in the future.

## 3. Materials and Methods

### 3.1. Chemicals

In this research, different chemicals were employed: (i) for the pH regulation, solutions of sodium hydroxide (NaOH, 1 mol L^−1^) and hydrochloric acid (HCl, 1 mol L^−1^) were used; (ii) for removing the extractives, ethanol (C_2_H_6_O, 80% *v*/*v*) was employed; (iii) zinc chloride (ZnCl_2_, solid form) was used as the activating agent; and (iv) different HCl solutions (10 mol L^−1^) were used for extracting the ZnCl_2_ after the pyrolysis. For the adsorption experiments, the propranolol hydrochloride (C_6_H_21_NO_2_.HCl_,_ M_w_ = 259.34 g mol^−1^) was used as adsorbed. The standard mother solution of propranolol hydrochloride (1 g L^−1^) was made using methanol (25 % *v*/*v*) to ensure that the propranolol hydrochloride was properly diluted. All the chemicals employed were acquired from Sigma-Aldrich-(Burlington, MA, USA), presenting analytical grades.

### 3.2. Instrumentation Techniques

In order to understand the proprieties of the precursor between the precursor material and AC after undergoing the carbonization process, a variety of techniques were utilized. These techniques included analyzing the structural, morphological, and chemical properties of the samples. One important factor examined was the specific surface area (S_BET_), which was obtained via the use of N_2_ adsorption/desorption isotherms obtained using ASAP 2020. Additionally, the total pore volume (VT) was determined by measuring the amount of N_2_ adsorbed on the material’s surface at a relative pressure (P/P_0_) of 0.99. Furthermore, the pore volumes were also calculated by measuring the volumes of N_2_ adsorbed at P/P_0_ values of 0.1 and 0.95. In order to evaluate the surface morphology of the samples, a scanning electron microscope, specifically a Vega 3 SB (Tescan, Brno, South Moravian, Czech Republic) instrument, was used. The working voltage was set to 10 kV and both pre-and post-pyrolysis samples were examined to evaluate any changes that occurred during the process. Fourier Transform Infrared (FTIR) spectra of the materials were attained by means of a Shimadzu IR-Prestige-21 FT-IR Spectrometer, which had a spectral range of 4500 to 500 cm^−1^. Finally, X-ray diffraction (XRD) was performed using a computer-controlled X-ray diffractometer (Rigaku, Miniflex 300, Tokio, Kantō, Japan) to identify the crystallographic structures of the samples. By using these various techniques to analyze the precursor material and resulting activated carbon, a more comprehensive understanding of the structural, morphological, and chemical differences between the two could be achieved. The pH of the solutions was determined by employing a Digimed pH meter (model DM 20, São Paulo, São Paulo, Brazil).

### 3.3. Preparation of Precursor Material and Activated Carbon

The preparation of the activated carbon involved three distinct steps: (i) acquiring and cleaning the precursor, (ii) mixing the agent and conducting pyrolysis, and (iii) removing the agent and drying the material. The residual fruits of *Bactris guineensis* were obtained from a local producer in Barranquilla, Colombia. Subsequently, the fruits were washed with tap water and the core was manually separated from the fruit. The core was then dried for 48 h at 328 K and ground using a knife mill (250 μm, 60 mesh). To ensure the presence of other organic matter alongside the core, extraction was performed using a solution of ethanol (80% *v*/*v* with a purity of 97%) until the extraction solution became colorless. The core powder was dried for 5 h at 328 K. The pyrolysis step commenced with a mixture of ZnCl_2_ and the core powder in a 1:1 ratio (15 g of each) with 5 mL of deionized water, forming a dark-colored paste. This mixture was dried for 48 h at 378 K, followed by further grinding to achieve particles smaller than 355 μm (45 mesh). The crushed particles were introduced into a quartz reactor installed in an oven with an N_2_ flow. The pyrolysis conditions were as follows: an N_2_ flow rate of 0.25 L min^−1^, a linear heating rate of 10 K min^−1^, a temperature set point of 923.15 K, and an operation time of 80 min. After the pyrolysis, the material was washed with a 10 mol L^−1^ HCl solution and distilled water until it reached a pH of 7. This step ensured the complete recovery of ZnCl2 and prevented interference with the subsequent adsorption experiments. Finally, the material was dried in an oven at 323.15 K for 8.3 h (500 min) and crushed to obtain particles smaller than 149 μm (100 mesh). The resulting material was labeled as activated carbon (AC) and set aside for characterization and adsorption experiments.

### 3.4. Propranolol Adsorption Experiments

All the samples were subjected to stirring in a thermostatic shaker (Brand: Marconi, model: MA093, Piracicaba, São Paulo, Brazil) at a constant speed of 150 rpm. To detect PROP in the liquid medium, a spectrophotometer (UV mini 1240, Shimadzu, Tokio, Kantō, Japan) was used, which was calibrated at a wavelength of 255 nm as the maximum absorption value of the compound. To ensure reliability, all the experiments were conducted with three repetitions and, after each test, the samples were centrifuged (Centribio 80-2B, Rio de Janeiro, Rio de Janeiro, Brazil) at 4000 rpm for 25 min to separate PROP/AC. To determine the ideal dosage and best pH of the solution, the study used two tests. In the dosage test, six Erlenmeyer flasks were filled with doses of 0.5, 0.6, 0.7, 0.8, 0.9, and 1 g L^−1^ and each flask was loaded with PROP solutions (25 mg L^−1^). The samples were stirred for 120 min at room temperature. In the pH test, eight Erlenmeyer flasks were used, with 25 mL of a PROP solution at a concentration of 25 mg L^−1^ being added to each flask. The pH of each flask was adjusted to 3, 4, 5, 6, 7, 8, 9, and 10 using NaOH and HCl solutions. The adsorption equilibrium was investigated for four temperatures (298, 308, 318, and 328 K) and four initial concentrations of PROP (25, 50, 75, and 100 mg L^−1^). To ensure equilibrium in the PROP/AC system, the flasks were shaken for a period of 5 h. The equilibrium time was determined by collecting samples at different sample times (ranging from 0 to 180 min) from the Erlenmeyer flasks containing 250 mL of solution with three different initial PROP concentrations (25, 50, and 75 mg L^−1^) at room temperature (298 K). The traditional batch adsorption model was used to determine the adsorption capacity of the samples given in the Supplementary Materials (S.2).

### 3.5. Adsorption Regeneration

Aiming to evaluate the application of the developed AC, regeneration tests were conducted. For this, AC loaded with PROP, obtained using the same conditions of the dosage and pH, was used. The cycle is described as follows: (i) loaded AC was put into contact with a 25 mL solution of methanol (80 % *v*/*v*); (ii) the solution was stirred for 180 min (same time internal as the adsorption kinetics); and (iii) the used AC was then put into contact with a solution of PROP (25 mg L^−1^) and stirred for 180 min once again. These 3 steps were considered a cycle, where, in this work, 7 cycles were performed to analyze the percentage of removal.

### 3.6. Adsorption Isotherm and Thermodynamics

In this research, various models were utilized to investigate the adsorption behavior of propranolol (PROP) onto activated carbon sourced from *Bactris guineensis* core. The models used included Freundlich [76], Langmuir [77], Redlich and Peterson [78], and Koble and Corrigan [79], which are thoroughly described in the Supplementary Materials (S.3). The Langmuir model assumes that the adsorbent surface is homogenous, whereas the Freundlich model characterizes the adsorption behavior of a solute on a heterogeneous adsorbent surface. On the other hand, the Redlich and Peterson model is a semi-empirical model based on the Langmuir model, which takes into account the effects of adsorbate–adsorbate interactions, and the Koble and Corrigan model recognizes the occurrence of pure species of the solute. The thermodynamic parameters were obtained using a method developed by Lima et al. [80,81], where the parameters from the best-fitted model to the equilibrium data were used, which is outlined in the Supplementary Materials (S.3 and S.4). These parameters provide crucial insights into the adsorption process’s spontaneity and nature and help in understanding the interaction mechanisms between the PROP and activated carbon.

### 3.7. Adsorption Kinetics

Several kinetic models were employed in this study to analyze the adsorption process. The pseudo-first-order (PFO) model, developed by Lagergren [82], assumes that the rate of adsorption is directly proportional to the number of available sites on the adsorbent surface. The pseudo-second-order (PSO) model, on the other hand, developed by Ho and McKay [83], considers that the ratio of accessible sites to their square is what determines the rate of adsorption. The general order (GO) model, presented by Liu and Shen [84], accounts for the nth power of unoccupied sites in the rate of adsorption. The linear driving force (LDF) model, proposed by Glueckauff [85], takes into account the theory that the rate of adsorption is proportional to the difference in concentration between the bulk solution and adsorbent surface. Detailed mathematical expressions for these models are provided in Supplementary Material S.5.

### 3.8. Estimation of Looped Parameters and Solution of the Differential Equation

The estimation of the model parameters and solution of the differential equation for the LDF model were obtained using Matlab scripting. The standard functions utilized in this study included particleswarm to provide an initial guess, nlinfit for the parameter estimation without boundary restrictions, lsqnonlin for the parameter estimation with boundary restrictions such as n ≤ 1, and ode15s for solving the partial differential equation. The quality of the model fit was assessed using various statistical indicators, which are available in Supplementary Material S.6.

## 4. Conclusions

This study explored the use of waste stone from corozo fruit as a source for obtaining activated carbon, which could provide a solution to the problem of the tons of waste generated annually. The activated carbon was prepared from the grounded and clean cores mixed with ZnCl_2_ (ratio of 1:1) at the temperature of 923.15 K for 80 min. The pyrolyzed material was washed with HCl (10 mol L^−1^) solution and distilled water until the water attained a neutral pH. The characterization of the material showed an adsorbent with a high specific surface area (1125 m^2^ g⁻^1^) and pore volume (3.241 × 10⁻^1^ cm^3^ g⁻^1^), with traditional functional groups (C-H, O-H, and C-O) with an amorphous nature. The adsorption isotherm showed endothermic behavior, where the maximum adsorption capacity of 112.9 mg g^−1^ was attained at 328.15 K. The isothermal data were best described by the Langmuir model, predicting a maximum adsorption capacity of 134 mg g^−1^ for the temperature of 328.15 K. The estimated thermodynamic proprieties corroborated the isothermal experimental results, presenting a standard Gibbs free energy change of −26.28 to −29.99 kJ mol^−1^, according to the system temperature. As for the standard enthalpy change, it was found to be +12.39 kJ mol^−1^, which is in agreement with the isothermal behavior. The adsorption kinetics for the propranolol were achieved in 30 min for the lower concentration and 60 min for the higher concentration, reaching adsorption capacities of 34.66 and 76.73 mg g^−1^. The general order model was found to be the proper one for describing the kinetical data, presenting an R^2^ _adj_ as high as 0.9935. As for the regeneration tests, it was found that the adsorbent could be recovered using a methanol solution (80 % *v*/*v*), retaining a percentage of removal above 75% up to six cycles. Overall, this research presents an alternative for the large-scale production of activated carbon from an abundant and renewable source, which could have implications for addressing environmental problems and developing effective adsorbents for the future.

## Figures and Tables

**Table 1:** Estimated model parameters and statistical indicators for the PROP/AC system.

	Temperature (K)			
Model	298.15	308.15	318.15	328.15
Langmuir				
q_mL_ (mg g^−1^)	121.4	141.6	135.9	134.7
K_L_ (L mg^−1^)	0.1556	0.1416	0.2044	0.2299
R^2^	0.9867	0.9876	0.9571	0.9551
R^2^_adj_	0.9733	0.9753	0.9142	0.9102
ARE (%)	7.964	8.143	15.41	15.83
MSR (mg g^−1^)^2^	28.28	30.51	112.0	119.4
BIC	18.16	18.54	25.04	25.36
Freundlich				
K_F_ ((mg g^−1^)(mg L^−1^)^−1/nF^)	25.06	27.24	27.96	28.24
1/nF (dimensionless)	0.4229	0.4501	0.4680	0.4744
R^2^	0.9656	0.9723	0.9355	0.9293
R^2^_adj_	0.9312	0.9447	0.8710	0.8585
ARE (%)	12.31	12.85	16.67	16.27
MSR (mg g^−1^)^2^	72.97	68.29	168.5	188.1
BIC	22.90	22.56	27.08	27.63
Redlich-Peterson				
K_RP_ (L g^−1^)	18.89	20.04	28.18	33.19
a_RP_ (L mg^−1^)	0.1556	0.1416	0.2142	0.2842
n_RP_ (dimensionless)	1.000	1.000	0.9900	0.9556
R^2^	0.9867	0.9876	0.9571	0.9553
R^2^_adj_	0.9467	0.9506	0.8285	0.8210
ARE (%)	7.964	8.143	15.40	15.74
MSR (mg g^−1^)^2^	42.42	45.77	168.0	178.4
BIC	20.16	20.54	27.04	27.34
Koble-Corrigan				
A (mg g^−1^) × (L mg^−1^)^nKC^	12.41	14.60	20.92	26.91
K_KC_ (L mg^−1^)^nKC^	0.1173	0.1199	0.1775	0.2191
n_KC_ (dimensionless)	1.361	1.298	1.353	1.204
q_KC_ (mg g^−1^)	105.8	121.7	117.9	122.8
R^2^	0.9891	0.9895	0.9583	0.9555
R^2^_adj_	0.9563	0.9579	0.8330	0.8221
ARE (%)	6.397	6.914	15.24	15.79
MSR (mg g^−1^)^2^	34.73	38.94	163.6	177.4
BIC	19.16	19.73	26.90	27.31
q_max,exp_ (mg g^−1^)	100.2	108.9	111.8	112.9

**Table 2:** Estimated thermodynamic values for the PROP/AC system.

T(K)	Ke × 10^−4^	ΔG^0^ (kJ mol^−1^)	ΔH^0^ (kJ mol^−1^)	ΔS^0^ (kJ mol^−1^ K^−1^)
298.15	4.035	−26.28	12.39	0.1290
308.15	3.672	−26.92		
318.15	5.301	−28.77		
328.15	5.962	−29.99		

**Table 3:** Kinetic parameters of PROP adsorption on activated carbon from *Bactris guineensis* stone.

Model	C_0_ (mg L^−1^)		
	25	50	75
Pseudo-first order			
q_1_ (mg g^−1^)	32.53	55.34	69.40
k_1_ (min^−1^)	0.1416	0.06094	0.05189
R^2^	0.9697	0.9178	0.9182
R^2^_adj_	0.9204	0.7898	0.7909
ARE (%)	6.307	12.92	13.56
MSR (mg g^−1^)^2^	4.033	33.10	50.82
BIC	14.29	33.23	37.09
Pseudo-second order			
q_2_ (mg g^−1^)	35.09	62.20	78.27
k_2_ × 10^3^ (g mg^−1^ min^−1^)	6.603	1.279	0.8580
R^2^	0.9980	0.9690	0.9649
R^2^_adj_	0.9946	0.9186	0.9080
ARE (%)	1.585	8.001	9.085
MSR (mg g^−1^)^2^	0.2686	12.48	21.82
BIC	−10.09	24.46	29.48
General order			
q_Av_ (mg g^−1^)	36.90	113.8	140.4
k_Av_ (min^−1^)	1.124 × 10^−3^	7.336 × 10^−14^	9.458 × 10^−14^
n_Av_ (dimensionless)	2.530	6.849	6.508
R^2^	0.9980	0.9690	0.9649
R^2^_adj_	0.9935	0.9023	0.8896
ARE (%)	0.9727	3.733	5.228
MSR (mg g^−1^)^2^	0.1283	3.650	8.898
BIC	−16.13	14.00	22.02
LDF			
qpred (mg g^−1^)	33.29	64.23	79.34
k_LDF_ × 10^4^ (s^−1^)	5.151	3.737	3.930
D_S_ × 10^9^ (cm^2^ s^−1^)	5.238	3.800	3.996
R^2^	0.9213	0.7882	0.8278
R^2^_adj_	0.9101	0.7580	0.8032
ARE (%)	10.26	25.47	29.62
MSR (mg g^−1^)^2^	9.159	74.58	93.65
BIC	20.87	39.75	41.80
q_exp_ (mg g^−1^)	34.66	62.92	76.73

## Data Availability

The corresponding author will provide the datasets produced and/or analyzed during the current work upon reasonable request. Data are available on request from the authors.

## Supplementary Materials

The following supporting information can be downloaded at: https://www.mdpi.com/article/10.3390/molecules28135232/s1, S.1: Batch adsorption equations; S.2: Isotherm models; S.3: Thermodynamic estimation; S.4:Kinetics models; S.5: Statistical evaluation; S.6: Figures; Figure S1: N_2_ adsorption and desorption isotherms for the activated carbon; Figure S2: Variation of the solution pH as function of the initial solution pH and representation of pHpzc and surface charges; Figure S3: Speciation diagram for the propranolol, according to the pH, prediction obtained from the Marvinsketch software.

## Sample Availability

Not available.
