# Supplementary material for: The Synthesis and Evaluation of Porous Carbon Material from Corozo Fruit (Bactris guineensis) for Efficient Propranolol Hydrochloride Adsorption
Source: Molecules. 2023 Jul 5;28(13):5232. doi: 10.3390/molecules28135232 (PMC10343689; doi:10.3390/molecules28135232)
Supplement: Supplementary file 1 [file molecules-28-05232-s001.zip › molecules-2465331-supplementary 2.pdf]

molecules MDPI

Article

Supplementary Materials

Dison Stracke Pfingsten Franco [1,*], Jordana Georgin [1,2,*], Claudete Gindri Ramos [1], Salma Martinez Eljaiek [1], Daniel Romero Badillo [1], Anelise Hoch Paschoalin de Oliveira [3], Daniel Allasia [2] and Lucas Meili [4]

[1] Department of Civil and Environmental, Universidad de la Costa, CUC, Calle 58# 55-66, Barranquilla, Atlántico 080002, Colombia
[2] Graduate Program in Environmental Engineering, Federal University of Santa Maria, Santa Maria 97105-900, RS, Brazil
[3] Graduate Program in Chemical Engineering, Federal University of Santa Maria, Santa Maria 97105-900, RS, Brazil
[4] Process Laboratory, Technology Center, Federal University of Alagoas 57072-870, Maceió, AL, Brazil
* Correspondence: francodison@gmail.com (D.S.P.F.); jordanageorgin89@gmail.com (J.G.)

S.1 Batch adsorption equations

The adsorption capacity at any time (q_t, mg g^{-1}), adsorption capacity at the equilibrium (q_e, mg g^{-1}), and removal percentage (R, %) values were calculated by the Equations (S2), (S3) and (S4), respectively:

$$q_t = (C_0 - C_t)\,\frac{V}{m} \tag{S1}$$

$$q_e = (C_0 - C_e)\,\frac{V}{m} \tag{S2}$$

$$R = \frac{(C_0 - C_t)}{C_0}\,100 \tag{S3}$$

Where: C_0 is the initial adsorbate concentration in the liquid phase (mg L^{-1}), C_e is the equilibrium concentration of the adsorbate in the liquid phase (mg L^{-1}), C_t is the adsorbate concentration in the liquid phase at any time (mg L^{-1}), m is the mass of adsorbent (g), and V is the volume of solution (L).

S.2 Isotherm models

The Langmuir model (Equation (4)), Freundlich model (Equation (S5)), and Koble-Corrigan model (Equation (S7)) are presented as follows:

$$q_e = q_L\,\frac{K_L C_e}{1+K_L C_e} \tag{S4}$$

$$q_e = K_F C_e^{1/n_f} \tag{S5}$$

$$q_e = \frac{K_{RP} C_e}{1+a_{RP} C_e^{n_{RP}}} \tag{S6}$$

$$q_e = \frac{A_{KC}(C_e)^{n_{KC}}}{1+K_{KC}(C_e)^{n_{KC}}} \tag{S7}$$

$$q_{KC}=\frac{A_{KC}}{K_{KC}} \quad (S8)$$

Where: q_L is the maximum adsorption capacity of Langmuir model (mg g^{-1}), K_L is the Langmuir constant (L mg^{-1}); K_F is the Freundlich constant ((mg g^{-1}) (mg $L^{-1})^{-1/n}$); $1/n_f$ (dimensionless) is the heterogeneity factor; K_{RP} is the Redlich-Peterson constant (L mg^{-1}), a_{RP} is the affinity coefficient (L $mg^{-1})^{nRP}$, n_{RP} is the Redlich-Peterson exponent; A_{KC} is the Koble-Corrigan constant ((mg g^{-1})(L $mg^{-1})^{nKC}$), K_{KC} is the Koble-Corrigan equilibrium constant ((L $mg^{-1})^{nKC}$), n_{KC} is the Koble-Corrigan exponent (dimensionless), q_{KC} is the Koble-Corrigan adsorption equilibrium (mg g^{-1}).

S.3 Thermodynamic estimation

The Gibbs free energy (ΔG^0, kJ mol^{-1}), enthalpy change (ΔH^0, kJ mol^{-1}), and entropy change (ΔS^0, kJ mol^{-1} K^{-1}) were calculated by the following Equations:

$$K_e = \frac{K_L M_W \gamma^{PROP}}{\Gamma} \quad (S9)$$

$$\Delta G^0 = -RTln(K_e) \quad (S10)$$

$$\Delta G^0 = \Delta H^0 - T\Delta S^0 \quad (S11)$$

$$ln(K_e) = \frac{\Delta S^0}{R} - \frac{\Delta H^0}{RT} \quad (S12)$$

where: K_e is the equilibrium constant (dimensionless), γ^{PROP} is the activity coefficient of the drug in solution (dimensionless, assuming $\gamma^{PROP} = 1$); Γ is the unitary activity coefficient of 1 mol L^{-1}); T is the temperature (K); R is the universal gas constant (8.31×10^{-3} kJ mol^{-1} K^{-1}).

S.4 Kinetic models

For describing the kinetics of adsorption the following models were adjusted to the experimental data: Pseudo-first order, Pseudo-second order, General order, Elovich, and Avrami

$$q_t = q_1 \, (1-e^{-k_1 t}) \quad (S13)$$

$$q_t = \frac{t}{\left(\frac{1}{k_2 q_2^2}\right) + \left(\frac{t}{q_2}\right)} \quad (S14)$$

$$q_t = \left(q_e - \frac{q_n}{\left[k_N.(q_n)^{n-1}.t.(n-1)+1\right]^{1/(1-n)}} \right) \quad (S16)$$

Where k_1 (min^{-1}), k_2 (g mg^{-1} min^{-1}), and k_N [(min^{-1})(g $mg^{-1})^{n-1}$] are the kinetics constants of pseudo-first-order, pseudo-second-order, and general order models, respectively; q_1, q_2, and q_n are the theoretical values for the adsorption capacity at the equilibrium for each

model (mg g^{-1}); n is the general order of General Kinetic model (dimensionless), t is contact time (min).

The linear driving force model (LDF) was also selected, where the adsorption rate can be expressed as:

$$\frac{d\bar{q}}{dt}=k_{LDF}\left(q_L\frac{K_LC_0\text{-}D_0\bar{q}}{1+K_LC_0\text{-}D_0\bar{q}}\text{-}\bar{q}\right) \quad (S17)$$

Furthermore, the diffusivity for a homogeneous particle can be estimated from the LDF kinetic lumped parameter:

$$D_S=\frac{{R_p}^2k_{LDF}}{15} \quad (S18)$$

R_p is the adsorbent particle radius, and D_s is the surface diffusivity (cm^2 s^{-1}).

S.5 Statistical evaluation

The determination coefficient (R^2) (Equation (S18)), adjusted determination coefficient (R^2_{adj}) (Equation (S19)), average relative error (ARE) (Equation (S20)), and mean square error (MSR) (Equation (S21)), Bayesian indicator criterion (BIC) are given by the following Equations:

$$R^2 = 1\text{-}\frac{\sum_{i=1}^{n}\left(y_{exp}\text{-}y_{pred}\right)^2}{\sum_{i=1}^{n}\left(y_{exp}\text{-}\bar{y}_{exp}\right)^2} \quad (S19)$$

$$R^2_{adj} = 1\text{-}\left(1\text{-}R^2\right)\frac{(n\text{-}1)}{(n\text{-}p\text{-}1)} \quad (S20)$$

$$ARE = \frac{100\%}{n}\sum_{i=1}^{n}\left|\frac{y_{exp}\text{-}y_{pred}}{y_{exp}}\right| \quad (S21)$$

$$MSR = \frac{1}{n\text{-}p}\sum_{i=1}^{n}\left(y_{exp}\text{-}y_{pred}\right)^2 \quad (S22)$$

$$BIC = nLn\left(\frac{RSS}{n}\right) + pLn(n) \quad (S23)$$

where: y_{exp} is the experimental value; y_{pred} is the predicted value; n is the number of experimental values; p is the number of parameters according to the model.

S.6 Figures

This section presents all the Figures called in the manuscript.

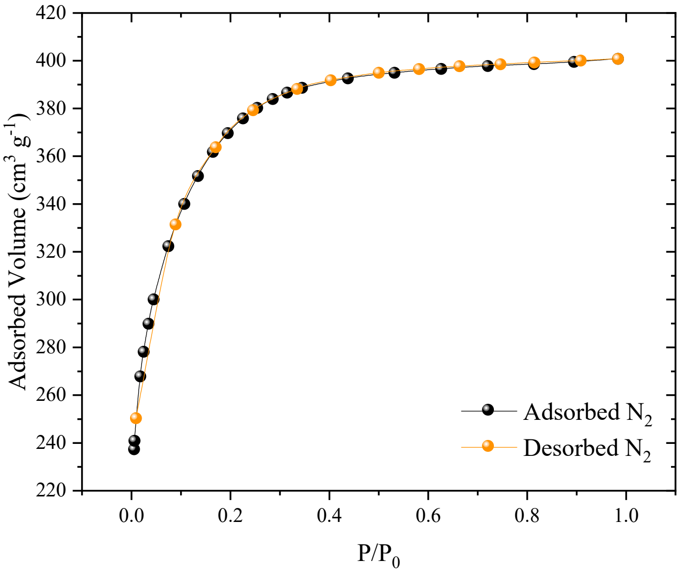

Figure S1. N_2 adsorption and desorption isotherms for the activated carbon.

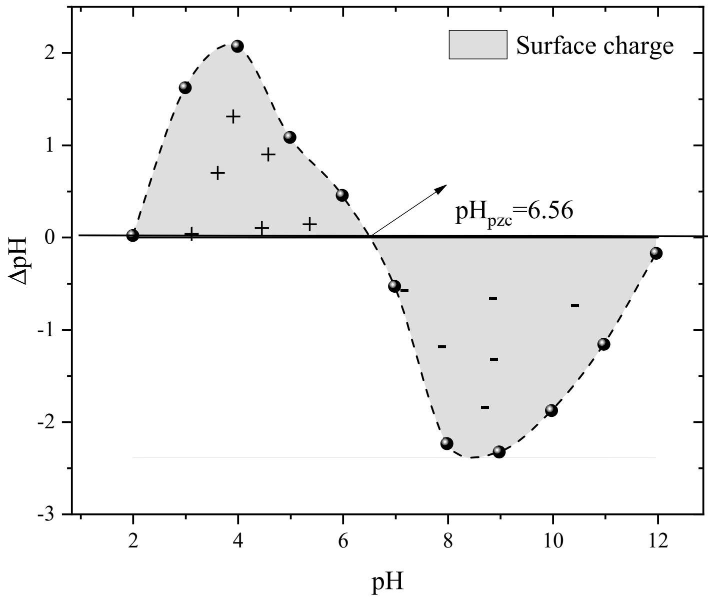

Figure S2. Variation of the solution pH as function of the initial solution pH and representation of pHpzc and surface charges

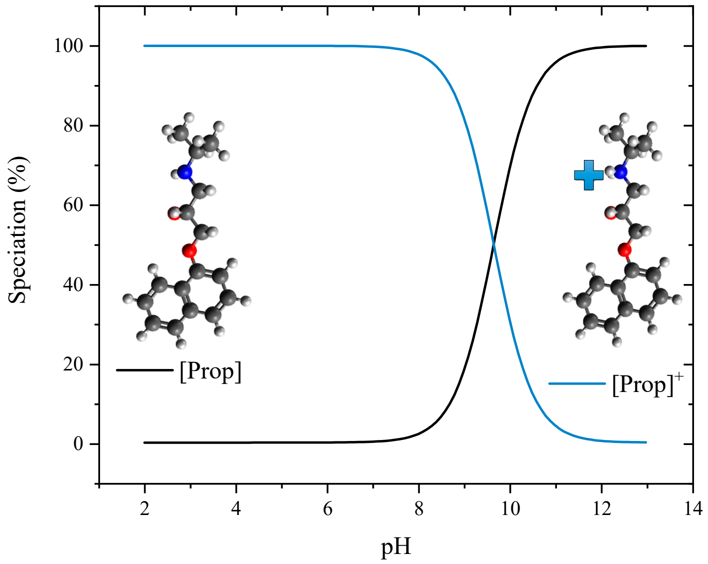

Figure S3. Speciation diagram for the propranolol, according to the pH, prediction obtained from the Marvinsketch software.

Disclaimer/Publisher's Note: The statements, opinions and data contained in all publications are solely those of the individual author(s) and contributor(s) and not of MDPI and/or the editor(s). MDPI and/or the editor(s) disclaim responsibility for any injury to people or property resulting from any ideas, methods, instructions or products referred to in the content.
